# The role of A-kinase anchoring proteins in cardiovascular diseases and recent advances

**DOI:** 10.3389/fcell.2025.1611583

**Published:** 2025-06-17

**Authors:** Xu Zhang, Feng Zhu, Zhaoqiang Xiao, Hegui Wang

**Affiliations:** ^1^ Department of Cardiology, Yijishan Hospital of Wannan Medical College, Wuhu, Anhui, China; ^2^ Department of Cardiology, Liyang Branch Hospital, The First Affiliated Hospital of Nanjing Medical University, Changzhou, Jiangsu, China; ^3^ Department of Cardiology, The First Affiliated Hospital of Nanjing Medical University, Nanjing, Jiangsu, China; ^4^ Department of Surgery, Fengcheng Hospital of Fengxian District, Shanghai, China

**Keywords:** A-kinase anchoring proteins, scaffold proteins, cellular microdomains, cardiovascular diseases, cardiovascular drugs

## Abstract

Cardiovascular diseases are a major global health concern, leading to high morbidity, mortality, and disability rates. Scaffold proteins, particularly A-kinase anchoring proteins (AKAPs), play a crucial role in signal transduction within the cardiovascular system. This review provides a comprehensive analysis of AKAPs’ involvement in the pathogenesis of cardiovascular diseases, emphasizing their key function in coordinating diverse signaling molecules, directing them to specific cellular microdomains, and minimizing signal interference. Disruptions in these interactions are linked to several cardiovascular disorders, such as cardiac hypertrophy, myocardial apoptosis, heart failure, arrhythmias, dysfunction in myocardial contraction and relaxation, and hypertension. Our goal was to explore the therapeutic potential of targeting the AKAP signaling pathway and offer new perspectives for the development and application of cardiovascular drugs that modulate AKAP signaling complexes.

## 1 Background

Cardiovascular diseases represent a major global health challenge, contributing considerably to both mortality and morbidity (Ong et al., 2018; Luo et al., 2021). A-kinase anchoring proteins (AKAPs) act as molecular scaffolds for a family of functionally related proteins that interact with various signaling molecules, including cyclic adenosine monophosphate (cAMP)-dependent protein kinases (PKAs). Furthermore, AKAPs associate with G-protein-coupled receptors, GTPases, kinases, phosphatases, phosphodiesterases, and cytoskeletal components, all of which are localized within distinct cytoskeletal microdomains. This strategic localization ensures that neighboring signaling complexes are insulated from other pathways, preserving specificity and minimizing potential crosstalk (Scott et al., 2013; Maric et al., 2021). The assembly of these multivalent signaling complexes allows AKAPs to integrate and coordinate signals from multiple pathways, enabling the precise regulation of complex cellular responses.

AKAPs are widely expressed in the heart and are critical to cardiac function. Notable examples include AKAP1 (D-AKAP1/AKAP121/AKAP149), AKAP5 (AKAP79/150/75), AKAP6 (mAKAPβ), AKAP7 (AKAP15/18), AKAP9 (Yotiao/AKAP350/450), AKAP10 (D-AKAP2), AKAP12 (Gravin), and AKAP13 (AKAP-Lbc) (Marin, 2020). A key feature of AKAPs is the structurally conserved domain responsible for PKA binding. This domain comprises a 14–18-residue amphipathic α-helix, which selectively interacts with the regulatory subunit of PKA, guiding its localization to specific subcellular regions where substrates are present (Per et al., 2012). Each AKAP, however, possesses a distinct subcellular targeting structure, with similar regions confined to homologous areas that bind to the RII dimer. PKA consists of two isoform types, I and II, each containing two regulatory subunits (RIα/β, RIIα/β) and two catalytic subunits (Cα, Cβ, or Cγ). According to the different regulatory subunits (RⅠ/RⅡ), PKA can be divided into two isoforms (PKAⅠ/PKAⅡ) (Christian et al., 2011; Liu et al., 2022; Bedioune et al., 2024). AKAPs are essential for compartmentalizing intracellular signal regulation, enabling precise control of site-specific signaling pathways. This regulation occurs through interactions with the regulatory subunits of PKA, particularly the RII subunits, which guide PKA to specific subcellular microdomains and modulate its substrate activity.

Some dual-specific AKAPs, or d-AKAPs, can simultaneously bind to both RI and RII (Table 1). RII subunits are typically localized to specific cellular sites, such as the plasma membrane, mitochondria, cytoskeleton, and centrosomes, whereas RI subunits tend to be more diffusely distributed (Kinderman et al., 2006; Omar and Scott, 2020). cAMP, as the second messenger activating PKA, is crucial for maintaining cellular physiological activities. In cardiomyocytes, the levels of cAMP are dynamically regulated by the balance between adenylate cyclases (ACs) and phosphodiesterases (PDEs). Upon activation of β-adrenergic receptors (β-ARs), the α subunit of G-proteins (Gas) is released within the target cells, which activates ACs, converting ATP to cAMP. This results in a rapid increase in intracellular cAMP levels and subsequent activation of PKA. In contrast, PDEs hydrolyze cAMP into 5′-AMP, reducing cAMP levels and consequently decreasing PKA activity (Liu et al., 2022; Zhang et al., 2024). The cAMP signaling pathway is compartmentalized. First, this second messenger is not uniformly distributed within the cell, and the spatial regulation of PDEs leads to varying cAMP concentrations across different subcellular compartments. PDEs regulate the localization, duration, and amplitude of cAMP signals within subcellular domains, controlling its diffusion to neighboring compartments, thereby preventing unnecessary PKA activation (Musheshe et al., 2018). Furthermore, AKAPs determine the subcellular localization of cAMP effectors by binding with PKA regulatory subunits and anchoring PKA to specific substrates. AKAPs can also interact with PDEs and phosphatases, providing local elements for signal termination. The spatial arrangement of these regulatory factors, effectors, and targets gives rise to the specific signaling of cAMP and governs the compartmentalized signaling mechanisms of AKAP complexes (Musheshe et al., 2018; Tomek and Zaccolo, 2023; Ripoll et al., 2024).

**TABLE 1:** Summary of AKAP complex features.

AKAPs	Alternative names	Selectivity	Associated proteins	Disease connection	References
AKAP1	D-AKAP1 AKAP121 AKAP149	PKA type I/PKA type II	CaN, NFATc3, NDUFS1, PKA BAD, Drp1 PTPD1, Src	Inhibition of cardiomyocyte hypertrophy and heart failure	Marin (2020), Abrenica et al. (2009), Paolillo et al. (2022), Diviani et al. (2011), Schiattarella et al. (2018), Liu et al. (2020)
				Inhibition of cardiomyocyte apoptosis	Qi et al. (2022), Qi et al. (2020), Perrino et al. (2010), Czachor et al. (2016), Kim et al. (2011), Schiattarella et al. (2016), Livigni et al. (2006)
AKAP5	AKAP79 AKAP150 AKAP75	PKA type II	PKA, PLN SERCA2a CaN, NFATc3 CaMKII, RyR2 PKC, CaV1.2 LQT8	Inhibition of cardiomyocyte hypertrophy and heart failure	De Windt et al. (2001), Li et al. (2014), Zhang et al. (2023), Wang et al. (2022), Zhu et al. (2022), Li et al. (2017), Bossuyt et al. (2011)
				Regulating cardiomyocyte apoptosis, myocardial contraction, and relaxation	Liu et al. (2022), Wang et al. (2022), Zeng et al. (2014), Bugger and Pfeil (2020), Tsai et al. (2012), Subramanian and Nikolaev (2023), Harvey and Hell (2013)
				Promote the occurrence of arrhythmias	Cheng et al. (2011)
				Regulating blood pressure	Navedo et al. (2008), Nystoriak et al. (2014), Pereira da Silva et al. (2022), Mercado et al. (2014), Chen et al. (2022)
mAKAPβ	AKAP6	PKA type II	CaN, NFATc3 PLCε, ERK5 PKA, PDE4D3 PKD, NCX1 RyR2	Promotion of cardiomyocyte hypertrophy and heart failure	Zhang et al. (2013), Zhang et al. (2011), Dodge-K et al. (2006), Li et al. (2019), Vergarajauregu et al. (2020), Dodge-Kafka et al. (2005), Nicol et al. (2001), Bedioune et al. (2018)
				Regulation of myocardial contraction and relaxation	Li et al. (2025), Turcotte et al. (2022), Schulze et al. (2003)
AKAP9	Yotiao AKAP350 AKAP450	PKA type II	PKA, AC PDE4D3 KCNQ1, PP1	Regulating cardiac arrhythmias	Marx et al. (2002), Chen and Kass (2011), Chen and Kass (2006), Morgat et al. (2024)
AKAP10	D-AKAP2	PKA type I/PKA type II	-	Associated with sinus arrhythmias, sinus pauses, and atrioventricular blocks	Kammerer et al. (2003), Colombe and Pidoux (2021), Tingley et al. (2007)
AKAP-Lbc	AKAP13 Ht31	PKA type II	PKA, PKC PKD, Gα12 RhoA, Bcl-2 SSH1L, cofilin2 Hsp20	Promotion of cardiomyocyte hypertrophy and heart failure	Appert-Collin et al. (2007), Kilian et al. (2021), Carnegie et al. (2008)
				Inhibition of cardiomyocyte apoptosis	Caso et al. (2017), Xiang et al. (2013), Edwards et al. (2012)
AKAP18	AKAP7 AKAP15	PKA type II	PKA, CaV1.2 PLN, SERCA2 CaMKII	Promoting cardiac contraction and regulating heartbeat	Carlson et al. (2022), Ercu and Klussmann (2018), Hulme et al. (2006), Lygren et al. (2007), Ahmad et al. (2015)

In cardiomyocytes, AKAP complexes play a critical role in maintaining and coordinating key cardiac functions by localizing to specific cellular sites. They regulate various physiological processes, including calcium cycling, excitation–contraction coupling, energy metabolism, transcriptional regulation, mitochondrial integrity, and action potential duration. Moreover, AKAPs play a crucial role in signaling pathways associated with pathophysiological conditions such as arrhythmias, cardiomyocyte hypertrophy, fibrosis, heart failure, and adaptive responses to hypoxia (Maric et al., 2021; Czepiel et al., 2022; Zhang et al.; Li et al., 2020; Delaunay et al., 2019; Carlson et al., 2022).

## 2 The role of AKAPs in cardiac hypertrophy development

**FIGURE 1:**
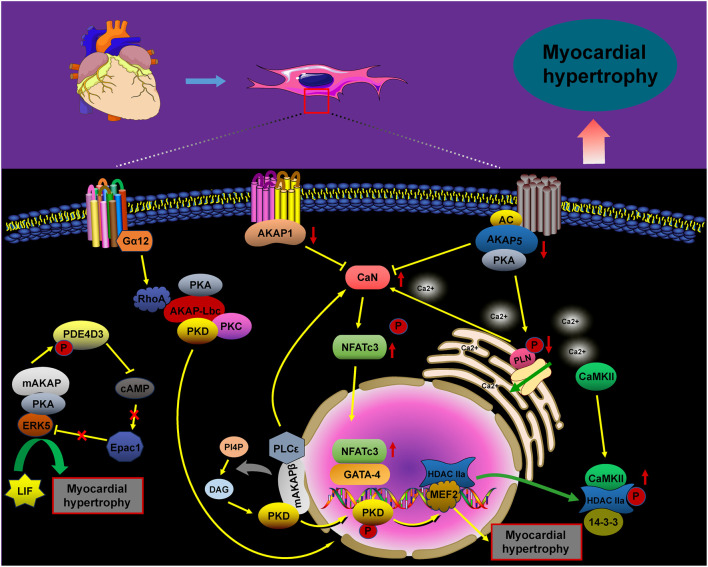
AKAP-mediated signaling in cardiomyocyte hypertrophy. The signaling pathways of AKAPs involved in cardiomyocyte hypertrophy, including the AKAP1/5/mAKAP/CaN/NFATc3, AKAP5/PKA/PLN, mAKAP/PLCε/PKD, mAKAP/PKA/PDE4D3, and Gα12/AKAP–Lbc/RhoA/PKD pathways, promote cardiomyocyte hypertrophy upon activation.

Pathological cardiac hypertrophy is characterized by heart dilation, triggered by various adverse factors. The mechanisms underlying this condition are complex and multifaceted. Notably, AKAPs play a critical role in the signaling pathways involved in cardiac hypertrophy (Figure 1). Specifically, AKAP1 acts as a negative regulator of cardiomyocyte hypertrophy through the calcineurin (CaN)/NFAT transcription factor 3 (NFATc3) pathway. In pathological conditions such as hypertension or chronic isoproterenol exposure, desensitization of β-adrenergic receptors (β-ARs) in cardiomyocytes leads to a decrease in AKAP1 gene transcription. As a result, the impaired AKAP1 protein is released into the cytoplasm by forming a complex with CaN, which dephosphorylates NFATc3, thereby triggering the gene expression associated with cardiac hypertrophy (Abrenica et al., 2009; Paolillo et al., 2022). Phosphorylated NFATc3 is predominantly inactive and localized to the cell membrane. However, genetic deletion of AKAP1 (AKAP1−/−) results in the release of active CaN. The availability of free CaN at the cell membrane promotes the dephosphorylation of NFATc3, facilitating its translocation from the membrane to the nucleus. This translocation activates cardiac hypertrophic genes, contributing to the progression of heart failure. Thus, AKAP1 may serve as a critical inhibitor of pathological cardiac hypertrophy (Paolillo et al., 2022; Diviani et al., 2011; Schiattarella et al., 2018).

AKAP5 shares significant homology with the CaN binding site in AKAP1 (Marin, 2020), and the AKAP5–CaN complex and its downstream effectors are crucial in the expression of pathological hypertrophic genes. Research has demonstrated that ΔAKAP5, which incorporates the CaN inhibitory structural domain of AKAP5, inhibits isoproterenol (ISO)-induced cardiac hypertrophy in genetically modified mice (De Windt et al., 2001). Li et al. found that carvedilol effectively reverses cardiac hypertrophy in AKAP5-deficient mice by normalizing the activity of cardiac CaN and calcium/calmodulin-dependent protein kinase II (CaMKII) (Li et al., 2014).

Following these findings, our study demonstrated that hypoxia/reoxygenation (H/R) reduces AKAP5 expression in cardiomyocytes. Concurrently, the activation of CaN and CaMKII promotes cardiomyocyte hypertrophy by modulating downstream molecular complexes (Zhang et al., 2023; Wang et al., 2022; Zhu et al., 2022; Dondi et al., 2024). The phosphorylation of phospholamban (PLN) by PKA, which is anchored by AKAP5, is essential for regulating intracellular calcium cycling. The phosphorylation or ablation of PLN, resulting in its dissociation from sarcoplasmic reticulum calcium ATPase 2a (SERCA2a), enhances calcium recycling through SERCA2a, thus reducing hypertrophic responses and arrhythmias in cardiomyocytes (Cheng et al., 2019; M et al., 2016; Bai et al., 2013; Fang et al., 2018; Koch et al., 2021).

Additionally, mAKAP plays a critical role in the development of cardiac hypertrophy. It functions as a docking platform for various signaling proteins, including CaN/NFATc3, phospholipase C epsilon (PLCε), and the kinase ERK5, all of which are directly anchored to the nuclear membrane of hypertrophic genes (Zhang et al., 2011; Dodge-K et al., 2006; Li et al., 2019). In addition to the classical hypertrophic pathway involving CaN/NFATc3, mAKAPβ anchors the PLCε complex at the nuclear membrane, facilitating the hydrolysis of phosphatidylinositol 4-phosphate (PI4P). The resulting hydrolysate, diacylglycerol (DAG), activates the hypertrophic pathway by promoting the activation of nuclear protein kinase D (PKD) and inhibiting myocardial hypertrophy considerably when mAKAPβ is depleted (Zhang et al., 2013; Vergarajauregu et al., 2020). Furthermore, mAKAP facilitates the phosphorylation of phosphodiesterase (PDE) 4D3 by anchoring PKA, which reduces local cAMP levels. This reduction weakens the inhibition of ERK5 activity mediated by Epac1, enhancing myocardial hypertrophy induced by leukemia inhibitory factor (LIF) (Dodge-Kafka et al., 2005; Nicol et al., 2001; Bedioune et al., 2018).

AKAP–Lbc, a scaffolding protein associated with protein kinase A (PKA) and protein kinase C (PKC), facilitates the activation of protein kinase D (PKD). This activation results in the phosphorylation of histone deacetylase 5 (HDAC5) and its translocation from the nucleus, thus enhancing the MEF2-mediated transcription of hypertrophic genes (Taglieri et al., 2014; He et al., 2020). Additionally, the Gα12–AKAP–Lbc–RhoA signaling pathway is likely involved in α1-adrenergic receptor (α1-AR)-induced hypertrophy of cardiomyocytes (Appert-Collin et al., 2007; Kilian et al., 2021).

## 3 The role of AKAPs in cardiomyocyte apoptosis

**FIGURE 2:**
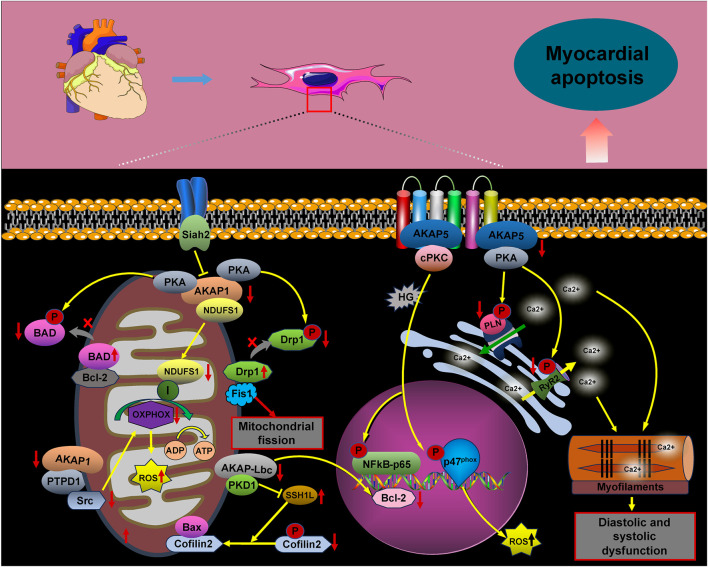
AKAP signaling in cardiomyocyte apoptosis. The signaling pathways involving AKAPs are crucial for cardiomyocyte apoptosis. Key pathways include AKAP5/PKA/PLN/RyR2, AKAP5/PKC/p47(phox), AKAP1/NDUFS1/OXPHOS, AKAP1/PKA/BAD, AKAP1/PKA/Drp1/Fis1, AKAP1/PTPD1/src, AKAP-Lbc/PKD1/SSH1L/Cofilin2, and AKAP-Lbc/PKD1/Bcl-2. Activation of these pathways promotes cardiomyocyte apoptosis.

Myocardial ischemia is a complex pathological condition resulting from reduced localized blood flow to tissues or organs. Although it prevents tissue necrosis, myocardial ischemia–reperfusion can induce metabolic disorders and cardiac dysfunction. Apoptosis, in particular, is a key pathological process (Chen et al., 2025; Han et al., 2024). AKAP complexes play a crucial role in regulating myocardial cell apoptosis (Figure 2).

Under physiological conditions, AKAP5 anchors PKA, facilitating the phosphorylation of ryanodine receptor 2 (RyR2) and PLN, which is crucial for maintaining intracellular calcium (Ca^2+^) cycling homeostasis. In contrast, mice deficient in AKAP5 exhibit reduced phosphorylation of RyR2 and PLN, leading to compromised Ca^2+^ cycling in response to adrenergic stimulation or pressure overload. This deficiency results in impaired myocardial diastolic and systolic function, along with significant cardiomyocyte apoptosis (Li et al., 2017). Our study showed a significant reduction in AKAP5 protein expression and a concomitant increase in apoptosis in H9C2 cells subjected to hypoxia/reoxygenation. Notably, activation of the PLN/SERCA2a signaling pathway following AKAP5 upregulation was linked to decreased apoptosis. Thus, AKAP5 may influence cardiomyocyte apoptosis through the PKA/PLN/SERCA complex (Wang et al., 2022). Additionally, under hyperglycemic conditions, AKAP5 and cPKC signaling complexes enhance anchoring at the plasma membrane and activate cPKC, promoting the phosphorylation of p47(phox) and the production of reactive oxygen species (ROS). This cascade leads to impaired myocardial diastolic function, apoptosis, and oxidative stress following hyperglycemic exposure (Zeng et al., 2014; Bugger and Pfeil, 2020; Tsai et al., 2012).

NADH-ubiquinone oxidoreductase subunit S1 (NDUFS1) protects against myocardial infarction and hypoxia-induced apoptosis associated with ROS in mitochondria. However, the deletion of AKAP1 impairs the mitochondrial translocation of NDUFS1, resulting in mitochondrial dysfunction, suppression of oxidative phosphorylation (OXPHOS), and increased mitochondrial ROS production. This cascade exacerbates cardiac myocyte apoptosis and contributes to the pathogenesis of diabetic cardiomyopathy (Qi et al., 2022; Qi et al., 2020). Furthermore, the downregulation of AKAP1 in response to pressure overload exacerbates mitochondrial dysfunction, increases ROS production, and promotes cardiomyocyte death (Perrino et al., 2010; Czachor et al., 2016). Research has shown that AKAP1 anchors protein kinase A (PKA) to various mitochondrial substrates, such as NDUFS4, enhancing the activity of the mitochondrial respiratory complex. Additionally, PKA exerts an anti-apoptotic effect by phosphorylating and inactivating the pro-apoptotic protein BAD, thereby preventing its association with Bcl-2 (Perrino et al., 2010; Harada et al., 1999; Haushalter et al., 2019; Affaitati et al., 2003; Gao et al., 2022). Kim et al. demonstrated that AKAP1, located on the mitochondrial membrane, plays a crucial role in inhibiting mitochondrial fission. This inhibition is facilitated by providing docking sites for PKA and dynamin-related protein 1 (Drp1), enabling the phosphorylation of Drp1 by PKA. Under ischemic hypoxic conditions, the expression and activity of Siah2 are upregulated, leading to the dysregulation of AKAP1. This dysregulation results in decreased Drp1 phosphorylation and increased interaction between Drp1 and Fis1, ultimately promoting mitochondrial fission and apoptosis in cardiomyocytes (Kim et al., 2011).

The observed reduction in AKAP1 levels under ischemic–hypoxic conditions may be linked to increased degradation of AKAP1, mediated by Siah2 (Schiattarella et al., 2016). Additionally, AKAP1 can direct src tyrosine kinase to the mitochondria through the action of protein tyrosine phosphatase (PTPD1). This targeting facilitates src-dependent tyrosine phosphorylation of mitochondrial substrates, thereby enhancing mitochondrial respiration and ATP synthesis (Perrino et al., 2010; Livigni et al., 2006). Mitochondrial dysfunction and apoptosis resulting from AKAP1 deficiency may be attributed to altered interactions between AKAP1, PKA, and src.

The literature indicates that AKAP–Lbc serves as a molecular scaffold, coordinating protective signaling pathways against doxorubicin (DOX)-induced cardiac cytotoxicity (Caso et al., 2017). Activation of PKD1, anchored by AKAP–Lbc, promotes the transcription of the anti-apoptotic protein Bcl-2 and inhibits the phosphatase SSH1L. This inhibition prevents the dephosphorylation of the actin-binding protein cofilin2, thus inhibiting the translocation of Bax to the mitochondria. As a result, this mitigates mitochondrial dysfunction, cytochrome C (CytC) release, and apoptotic cell death (Caso et al., 2017; Xiang et al., 2013). Additionally, localized increases in cyclic adenosine monophosphate (cAMP) due to β-adrenergic receptor stimulation activate PKA, anchored by AKAP–Lbc. This activation enhances the phosphorylation of the 20-kDa heat shock protein (Hsp20) at the Ser16 site, preventing cardiomyocyte apoptosis (Edwards et al., 2012).

## 4 The role of AKAPs in cardiac arrhythmias

**FIGURE 3:**
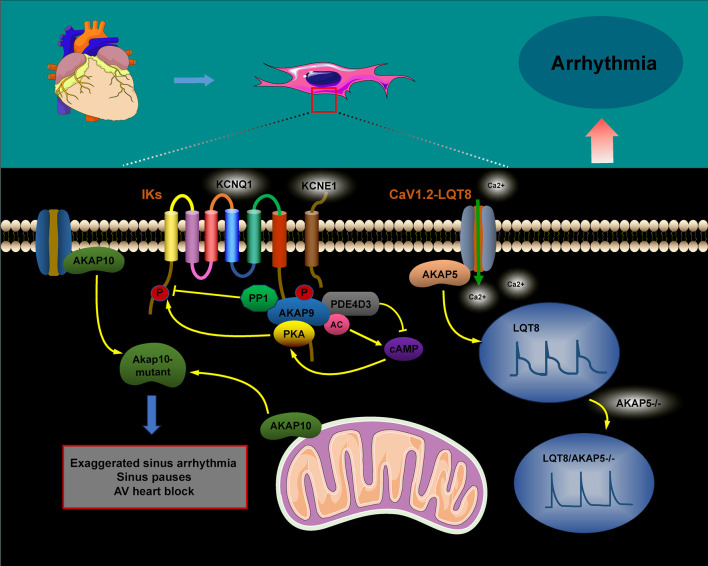
The role of AKAPs in signaling pathways during cardiac arrhythmias. The AKAP9/AC/PDE4D3/PKA and AKAP9/PP1 complexes regulate KCNQ1 phosphorylation, thus controlling the activity and current kinetics of IKs. Mutations in KCNQ1 within this complex are associated with LQT1. AKAP5 enhances calcium influx, prolongs action potential duration, and promotes arrhythmogenesis by increasing the coupling and gating of the CaV1.2–LQT8 channel, while AKAP5 ablation corrects the CaV1.2–LQT8-related arrhythmias. AKAP10 mutations can result in sinus arrhythmia, sinus arrest, and atrioventricular conduction blocks.

In the cardiac system, the AKAP macromolecular complex coordinates the phosphorylation of various channel proteins, including RyR2 calcium channels, L-type calcium channels, and potassium channels (IKs). Mutations in these channels have been linked to inherited arrhythmia syndromes, such as long QT syndrome (LQT) and catecholaminergic polymorphic ventricular tachycardia (Chen et al., 2007; Hegyi et al., 2021; Wu and Larsson, 2020). The sympathetic nervous system tightly regulates the activation of IKs, with AKAPs playing a crucial role in cardiac repolarization. Studies have shown that AKAP9 (Yotiao) forms a macromolecular complex with the α-subunit of the IK potassium channel (KCNQ1), the regulatory subunit of PKA type II (RII), and protein phosphatase 1 (PP1). Additionally, AKAP9 can activate PKA by modulating cAMP levels through interactions with adenylyl cyclase (AC) and phosphodiesterase 4D3 (PDE4D3). Activated PKA then facilitates the phosphorylation of serine 43 (S43) on AKAP9 and promotes the phosphorylation of serine 27 (S27) in the amino-terminal region of KCNQ1. The removal of phosphorylation at the AKAP9 S43 site considerably impairs PKA-induced voltage-dependent activation of IKs, altering these dynamics. Conversely, AKAP9’s binding to PP1 leads to the dephosphorylation of KCNQ1, and mutations within this complex have been implicated in type 1 long QT syndrome (LQT1), a potentially fatal inherited arrhythmia syndrome (Marx et al., 2002; Chen and Kass, 2011; Chen and Kass, 2006; Morgat et al., 2024) (Figure 3).

Timothy syndrome (TS), also referred to as long QT syndrome type 8 (LQT8), is a rare pediatric disorder caused by the G406R mutation in the CaV1.2 channel. This mutation disrupts the normal inactivation of the channel, resulting in a prolonged influx of Ca^2+^ during the action potential (AP). This predisposes individuals to life-threatening arrhythmias (Drum et al., 2014; Pitt et al., 2021). CaV1.2 channels are crucial for excitation–contraction coupling in the heart, as they considerably influence the AP waveform. They play an essential role in the heart’s excitation–contraction mechanism. In the case of TS (LQT8), the interaction between the anchoring protein AKAP5 and the CaV1.2–LQT8 channel forms a complex that enhances calcium influx, prolongs the AP duration, and promotes arrhythmogenesis. This occurs through stabilization of the open conformation and facilitation of the gating of the CaV1.2–LQT8 channel. Interestingly, AKAP5 ablation has been shown to rectify the pathological gating of the CaV1.2–LQT8 channel, helping mitigate the development of arrhythmias (Cheng et al., 2011).

AKAP10 is a bispecific A-kinase anchoring protein primarily localized in the mitochondria, cytoplasm, and plasma membrane, where it plays a crucial role in regulating heart rate in both mice and humans. A functional single nucleotide polymorphism (SNP) in AKAP10 has been identified, involving the substitution of isoleucine (Ile) at position 646 with valine (Val). Individuals carrying this SNP exhibit an increased heart rate, reduced heart rate variability, and an higher risk of cardiac arrest and sudden death (Kammerer et al., 2003; Colombe and Pidoux, 2021; Tingley et al., 2007). Tingley et al. demonstrated that mutations in AKAP10 increased the sensitivity of cardiomyocytes to cholinergic signaling, contributing to arrhythmia development. Mice with AKAP10 mutations exhibited significant sinus arrhythmias, sinus pauses, and atrioventricular blocks. These mice experienced sinus pauses with junctional escape beats 40 times more frequently and atrioventricular block 15 times more frequently than wild-type (WT) mice (Tingley et al., 2007). Additionally, a correlation was proposed between the 1936A > G AKAP10 variant and the corrected QT interval (QTc) in a cohort of European-descent newborns (Loniewska et al., 2015).

## 5 The role of AKAPs in heart failure

**FIGURE 4:**
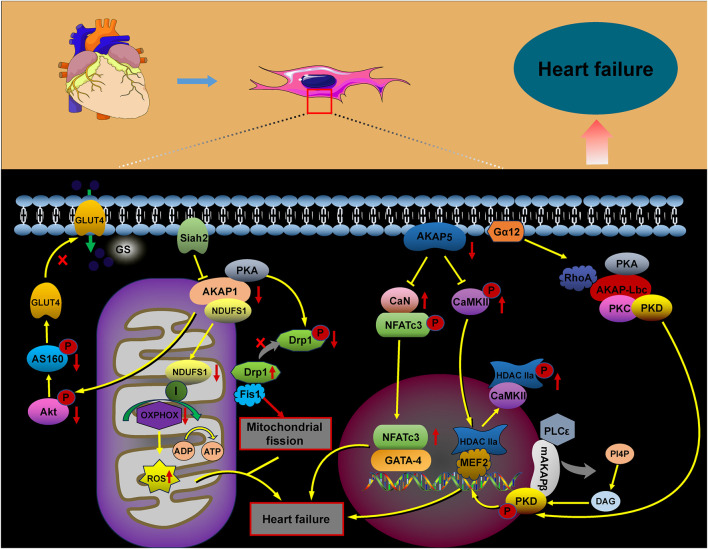
Role of AKAP signaling in heart failure. The AKAP signaling pathways involved in heart failure, including the AKAP1/Akt/AS160, AKAP1/PKA/Drp1/Fis1, AKAP1/NDUFS1/OXPHOS, AKAP5/CaN/NFATc3, AKAP5/CaMKII/HDAC, AKAP-Lbc/PKD/HDAC, and mAKAPβ/PLCε/PKD pathways, contribute to heart failure progression through their activation.

Heart failure is a progressive, often fatal condition marked by a decline in the heart’s ability to pump blood efficiently. Factors such as myocardial infarction, hypertension, congenital heart disease, and chronic activation of neurohumoral factors and cytokines contribute to its onset. The progression of heart failure is further aggravated by cardiomyocyte hypertrophy, apoptosis, and fibrotic remodeling. The AKAP complex plays a significant role in the pathophysiology of heart failure (Figure 4), with protein kinase B (Akt), a crucial signaling molecule involved in cardiomyocyte growth and diastolic function, influencing its progression through various signaling pathways. Research suggests that Akt can promote the translocation of glucose transporter 4 (GLUT4) to the plasma membrane by activating the substrate AS160. This action enhances glucose uptake, helping to prevent diabetic cardiomyopathy and heart failure (Hou et al., 2019; Shu et al., 2021). In AKAP1 knockout mice subjected to transverse aortic constriction (TAC), the absence of Akt activation accelerated cardiomyocyte death and worsened the progression of heart failure (Marin, 2020; Schiattarella et al., 2018). Abnormalities in mitochondrial metabolism, impaired oxidative phosphorylation (OXPHOS), excessive ROS production, and dysregulated mitochondrial dynamics have all been implicated in the development of heart failure (Wu et al., 2022). Specifically, downregulation of AKAP1 reduces PKA localization to the mitochondria, resulting in decreased inhibitory phosphorylation of Drp1 at serine 637. This promotes mitochondrial fission, leading to increased cytoplasmic ROS production, which contributes to cardiac hypertrophy and the progression of heart failure (Liu et al., 2020).

Abnormal changes in cardiomyocyte properties are strongly associated with heart failure. These alterations include the reactivation of fetal gene programs, disruptions in calcium handling and energy metabolism, abnormal protein synthesis, and sarcomere reorganization. Such modifications impair myocardial contractility, promote cardiomyocyte apoptosis, and exacerbate the progression of heart failure. A-kinase anchoring proteins play a critical role in coordinating the signaling pathways involved in these processes (Cibi et al., 2020; Subramanian and Nikolaev, 2023). Heart failure is marked by increased activation of the CaN–NFAT signaling pathway. AKAP5 plays a cardioprotective role by modulating the pathological signaling triggered by β-adrenergic receptors (β-ARs) and CaN. Notably, the absence of AKAP5 in murine models leads to a considerable rise in CaN and CaMKII activity, which is closely associated with the onset of age-related cardiac hypertrophy, ventricular dilation, and the progression of heart failure (Li et al., 2014; Li et al., 2017; Bossuyt et al., 2011). The mAKAPβ/PLCε/PKD and AKAP-Lbc/PKD/HDAC5 pathways play a critical role in regulating myocardial hypertrophy, considerably contributing to the progression of heart failure (Zhang et al., 2013; Vergarajauregu et al., 2020; He et al., 2020; Appert-Collin et al., 2007; Kilian et al., 2021; Carnegie et al., 2008).

## 6 The role of AKAPs in myocardial contraction and relaxation

AKAPs are integral to the signaling pathways of various hormones and neurotransmitters within the heart, facilitating excitatory–contractile coupling and intracellular calcium cycling in cardiomyocytes in response to β-AR stimulation. In this signaling pathway, AKAPs anchor PKA and regulate the phosphorylation of key substrate proteins, including L-type calcium channels (LTCC, Cav1.2), ryanodine receptors (RYRs), PLN, troponin I (cTnI), and cardiac myosin-binding protein C (cMyBP-C) (Carlson et al., 2022; Wang et al., 2022; Li et al., 2017; Rababa’h et al., 2014; Pallien and Klussmann, 2020).

During the excitation–contraction coupling (ECC) process, the transient opening of L-type calcium channels (LTCCs) in the transverse tubules and surface sarcoplasmic membrane causes a localized increase in intracellular calcium concentration ([Ca^2+^]i). This increase activates ryanodine receptor 2 (RyR2) in the sarcoplasmic reticulum (SR) via calcium-induced calcium release (CICR). Consequently, a calcium transient occurs, leading to an overall increase in [Ca^2+^]i and subsequent contraction of the cardiomyocyte. Following this, LTCC and RyR2 rapidly inactivate through a calcium-dependent mechanism, halting further calcium release from the SR. This process enables the sarcoplasmic/endoplasmic reticulum calcium ATPase 2a (SERCA2a) to recycle the released calcium before the next heartbeat. SERCA2a plays a critical role in regulating myocardial calcium cycling by facilitating the reuptake of considerable amounts of cytoplasmic calcium into the SR (Rhana et al., 2024; Jiang et al., 2025).

**FIGURE 5:**
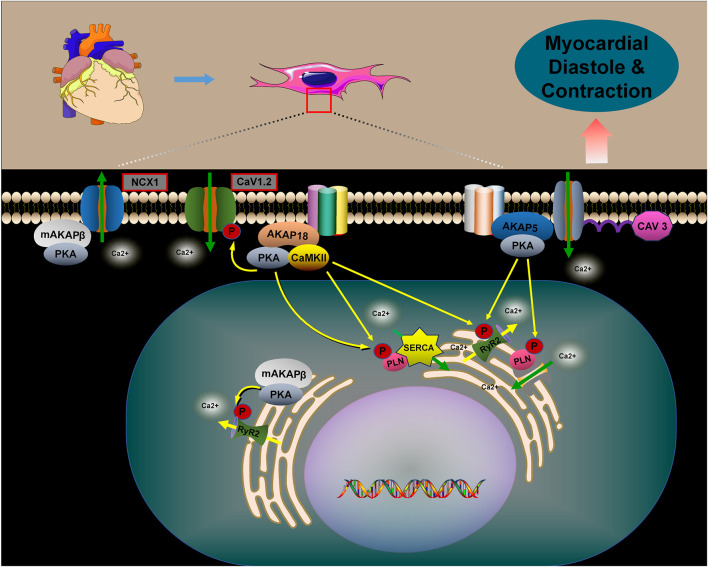
The role of AKAPs in regulating myocardial contraction and relaxation. The role of AKAPs in regulating myocardial contraction and relaxation is pivotal. Specifically, AKAP5, AKAP18, and mAKAPβ regulate cellular calcium cycling, cardiac contraction, and relaxation by forming complexes with PKA, CaV1.2, PLN, and RyR2. In the absence of AKAPs, disruptions in calcium cycling occur within cardiomyocytes, leading to abnormalities in myocardial contraction and relaxation. Furthermore, mAKAPβ plays a crucial role by interacting with NCX1, thereby promoting PKA-dependent activation of NCX1. This interaction facilitates the efflux of Ca2+, thus influencing myocardial contraction and relaxation.

PLN negatively regulates SERCA2a activity by binding to it and reducing its calcium affinity (MacLennan and Kranias, 2003; Weber et al., 2021; Ren et al., 2024). Phosphorylation of PLN, mediated by AKAPs in conjunction with PKA, causes PLN to dissociate from SERCA2a. This process improves the calcium recycling by SERCA2a into the endoplasmic reticulum, reducing cytoplasmic calcium accumulation and promoting cardiomyocyte diastole. The phosphorylation of these calcium-related proteins is regulated by multiple AKAPs (Subramanian and Nikolaev, 2023; Szentesi et al., 2004; Papa et al., 2022) (Figure 5).

AKAP5 macromolecular complexes consist of β-ARs, PKA, CaN, PLN, RyR2, and others. PKA-mediated phosphorylation regulates the activities of CaV1.2, PLN, and RyR2, which are associated with CaV3. AKAP5 anchors PKA to CaV1.2, PLN, and RyR2, promoting Ca^2+^ release through the activation of CaV1.2. This activation in turn stimulates RyR2 and PLN, playing a crucial role in myocardial contraction and relaxation (Liu et al., 2022; Harvey and Hell, 2013; Xiao et al., 2005). Studies have shown that genetically engineered mice lacking AKAP5 exhibit reduced calcium release from RyR2 channels and impaired calcium recycling by PLN/SERCA2a. This dysfunction results from diminished phosphorylation of RyR2 and PLN by the AKAP5/PKA complex, disrupting calcium cycling in cardiomyocytes and leading to abnormal myocardial contraction and relaxation (Li et al., 2017; Subramanian and Nikolaev, 2023).

AKAP18α is a membrane-associated scaffolding protein that enhances calcium currents by facilitating PKA-dependent phosphorylation of serine 1928 in CaV1.2 channels, thus promoting cardiac contraction (Ercu and Klussmann, 2018; Hulme et al., 2006). In rat hearts, AKAP18δ forms a supramolecular complex with PKA, PLN, and SERCA2, anchoring PKA to phosphorylate PLN in response to adrenergic stimulation. This process regulates SERCA2-mediated Ca^2+^ reuptake into the SR (Lygren et al., 2007). AKAP18δ is likely crucial for regulating the heartbeat by modulating the Ca^2+^ frequency-dependent activation of CaMKII at the SERCA2–PLN complex and RyR channels (Carlson et al., 2022). In the human heart, AKAP18γ promotes PKA-mediated phosphorylation of PLN, leading to its dissociation from SERCA2, which activates ATPase and enhances Ca^2+^ reuptake into the SR (Ahmad et al., 2015). The muscle-selective A-kinase anchoring protein, mAKAPβ, interacts with RyR2 at the SR, promoting PKA-mediated phosphorylation of the receptor. This modification enhances channel opening, facilitating the release of Ca^2+^ from the SR into the cytoplasm (Li et al., 2025; Turcotte et al., 2022). Additionally, mAKAPβ interacts with the sodium/calcium exchanger protein NCX1 at the sarcolemmal membrane, facilitating PKA-dependent activation of NCX1. This leads to an increased Ca^2+^ efflux (Schulze et al., 2003).

## 7 The role of AKAPs in hypertension pathogenesis

**FIGURE 6:**
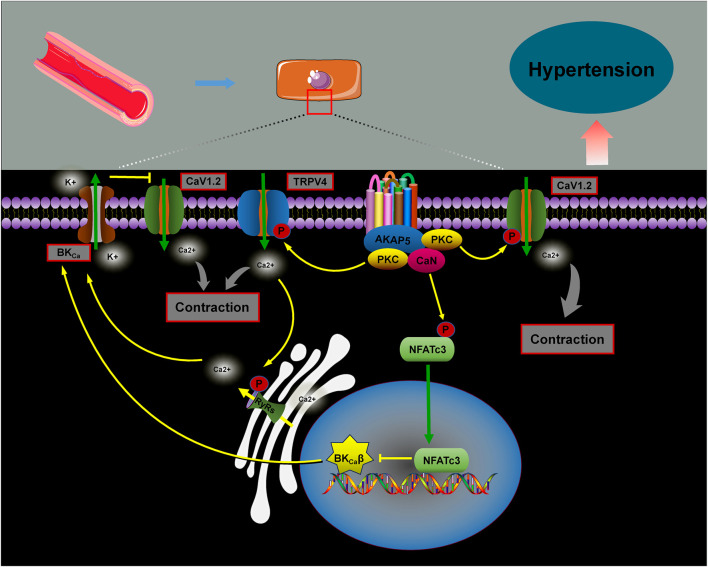
The role of AKAPs in blood pressure regulation. AKAP5 facilitates calcium influx by anchoring PKC to phosphorylate Cav1.2 channels, which activates the CaN/NFATc3 complex. This reduces the expression of the β1 subunit of BKCa, inhibiting potassium efflux, promoting vascular contraction, and raising blood pressure. Additionally, studies show that the AKAP5–PKC complex interacts with the TRPV4 calcium channel, promoting RyRs–BKCa coupling and activating the BKCa channel, thereby suppressing blood pressure elevation.

Hypertension is a major risk factor for the development of cardiovascular, cerebrovascular, and renal diseases. Its etiology is multifactorial, with vascular dysfunction playing a central role (Navedo et al., 2008). Changes in vasoconstrictive properties and increased arterial remodeling contribute to the progression of conditions like chronic hypertension, atherosclerosis, and heart failure (Touyz et al., 2018). Research has demonstrated that AKAPs are vital in regulating vascular integrity and peripheral arterial vasoconstriction by integrating and processing various signal transduction pathways, which are essential for maintaining blood pressure homeostasis (Nystoriak et al., 2017; Prada et al., 2020; Ottolini et al., 2020) (Figure 6).

AKAP5 is a key scaffolding protein involved in regulating blood pressure. It facilitates calcium influx through PKC activation of the voltage-dependent calcium channel CaV1.2, promoting cellular contraction and increasing vascular tone. In contrast, the absence of AKAP5 disrupts the PKC-mediated targeting of CaV1.2, leading to reduced calcium release and decreased vascular tone (Navedo et al., 2008). Under hyperglycemic conditions, AKAP5 anchors PKC to phosphorylate the Cav1.2 channel, enhancing calcium ion influx and activating the CaN/NFATc3 complex. This results in reduced expression of the β1 subunit of the large-conductance calcium-activated K^+^ channel (BKCa), inhibiting K^+^ efflux, promoting vasoconstriction, and raising blood pressure (Nystoriak et al., 2014; Pereira da Silva et al., 2022). However, evidence suggests that the AKAP5–PKC complex also interacts with transient receptor potential vanilloid 4 (TRPV4) calcium channels, facilitating the coupling of RyRs to BKCa channels. This interaction helps inhibit the increase in blood pressure (Mercado et al., 2014; Chen et al., 2022).

## 8 Conclusion

AKAPs are ubiquitously expressed in the cardiovascular system, anchoring various signaling molecules to multiprotein complexes. As dynamic hubs for multiple signaling pathways, AKAPs are essential for maintaining the homeostasis and functionality of the cardiovascular system. Disruptions in the interactions between AKAPs and their associated molecules are closely linked to the pathophysiology of cardiovascular diseases, including heart failure, cardiomyocyte apoptosis and hypertrophy, myocardial contractile and diastolic dysfunction, arrhythmias, and hypertension. Preliminary drug development and therapeutic strategies have begun to target AKAPs as potential interventions for cardiovascular diseases. One such agent, St-Ht31, is a peptide inhibitor of AKAPs that disrupts the interaction between AKAPs and PKA by mimicking the RIIα binding domain of PKA. This disruption results in decreased phosphorylation of the PKA substrate, RyR2 (Marx et al., 2000; Soni et al., 2014; McConnell et al., 2009). Hyperphosphorylation of RyR2 has been implicated in the pathogenesis of various cardiac dysfunctions, including myocardial systolic and diastolic dysfunction, arrhythmias, and heart failure (Marx et al., 2000; Belevych et al., 2013; Do and Knollmann, 2025; Shan et al., 2010). Despite its widespread use in fundamental research, the peptide inhibitor St-Ht31 has multiple drawbacks (Troger et al., 2012). St-Ht31 lacks specificity, targeting multiple AKAP isoforms and potentially interfering with distinct AKAP-mediated signaling pathways, which complicates the interpretation of its inhibitory effects. Moreover, it has shortcomings such as limited cellular uptake and a short biological half-life. Thus, further investigation is necessary to fully assess St-Ht31’s impact on cardiac conditions. In contrast, the small molecule inhibitor FMP-API-1 is better suited for cellular and animal studies due to its stability. FMP-API-1 has shown promise in disrupting the AKAP–PKA interaction and enhancing myocardial contractility in rats. However, the enhanced myocardial contractility observed with FMP-API-1 may also result from its activation of PKA (Christian et al., 2011; Troger et al., 2012). Recent advances in gene regulation technologies, such as CRISPR-Cas9 gene editing, RNA interference, and viral vector-mediated gene therapy, have shown considerable promise in cardiovascular disease research by enabling precise targeting of AKAPs (Carlson et al., 2022; Caso et al., 2017; Ibarrola et al., 2018; Guo et al., 2015; Mayers et al., 2010; You et al., 2022; Xu et al., 2025). The crucial role of AKAPs in cardiovascular diseases has been consistently validated. Ongoing and future research into AKAP complexes has the potential to provide novel insights that could overcome the limitations of conventional therapies and inform the development of molecularly targeted drugs for the treatment of cardiovascular diseases.

## Glossary

AC: adenylate cyclase
ADP: adenosine diphosphate
AKAP: A-kinase anchoring protein
Akt: protein kinase B
AS160: Akt substrate of 160 kDa
ATP: adenosine triphosphate
BAD: Bcl-2 antagonist of cell death
Bax: Bcl-2-associated X protein
Bcl-2: anti-apoptotic B cell leukemia/lymphoma 2
BKCa: large conductance calcium-activated potassium channel
CaMKII: Ca^2+^/calmodulin-dependent protein kinase type II
cAMP: cyclic-3′,5′-adenosine monophosphate
CaN: calcineurin
CaV1.2: voltage-gated calcium channels
Cav3: caveolin 3
DAG: diacylglycerol
Drp1: dynamin-related protein 1
Epac1: exchange protein activated by cAMP 1
ERK5: extracellular signal-regulated kinase 5
Fis1: mitochondrial fission 1 protein
GLUT4: glucose transporter type-4
Gα12: G protein subunit alpha 12
HDAC: histone deacetylase
IKs: slow component of the delayed rectifier K^+^ current
KCNQ1: potassium voltage-gated channel subfamily Q member 1
LIF: leukemia inhibitory factor
LQT8: type 8 long QT syndrome
mAKAP: muscle-specific A kinase anchoring protein
MEF2: myocyte enhancer factor 2
NCX1: sodium/calcium exchanger
NDUFS1: ubiquinone oxidoreductase core subunit S1
NFAT: nuclear factor of activated T cells
NFkB: nuclear factor kappa B
OXPHOS: oxidative phosphorylation
p47(phox): a component of NADPH oxidase
PDE4D3: phosphodiesterase-4D3
PI4P: phosphatidylinositol 4-phosphate
PKA: protein kinase A
PKC: protein kinase C
PKD: protein kinase D
PLCε: phospholipase Cε
PLN: phospholamban
PP1: Ser/Thr protein phosphatase 1
PTPD1: protein-tyrosine phosphatase D1
RhoA: Ras homolog gene family member A
ROS: reactive oxygen species
RyR2: ryanodine receptor 2
RyRs: ryanodine receptor
SERCA: sarco/endoplasmic reticulum Ca^2+^-ATPase
Siah2: seven in absentia homolog 2
Src: a non-receptor tyrosine kinase protein
SSH1L: slingshot-1L
TRPV4: transient receptor potential vanilloid 4
